# A Case of Urinary Tract Infection Resulting From Voiding Dysfunction Caused by Sacral Herpes Zoster Involving the Left Buttock

**DOI:** 10.7759/cureus.115550

**Published:** 2026-08-31

**Authors:** Eri Uchiyama, Fumiaki Taga, Akira Shimizu, Kengo Furuichi, Takayuki Yamaguchi

**Affiliations:** 1 Department of Dermatology, Kanazawa Medical University, Ishikawa, JPN; 2 Department of Nephrology, Kanazawa Medical University, Ishikawa, JPN

**Keywords:** acyclovir, antibiotics, dysuria, herpes zoster, immunosuppression, kidney transplantation, prednisone, pyelonephritis, sacral pulp area, urinary retention

## Abstract

Herpes zoster-associated voiding dysfunction is an uncommon complication but may occur when the sacral dermatomes are involved. In immunocompromised patients, including kidney transplant recipients receiving immunosuppressive therapy, urinary retention may predispose patients to secondary urinary tract infections such as pyelonephritis. Prompt recognition and appropriate management are therefore essential.

We report the case of a woman with a history of living-donor kidney transplantation who developed urinary retention and pyelonephritis following herpes zoster involving the buttock. She had been receiving maintenance immunosuppressive therapy with methylprednisolone, mizoribine, and cyclosporine. After the diagnosis of herpes zoster, antiviral therapy was initiated. Several days later, she developed dysuria caused by urinary retention, which was successfully managed with corticosteroid therapy and temporary urinary catheterization. However, she subsequently developed fever and was diagnosed with acute pyelonephritis on hospital day 7. Intravenous antibiotic therapy resulted in gradual clinical improvement, and inflammatory markers and urinary findings normalized by hospital day 15.

This case highlights that sacral herpes zoster can cause neurogenic bladder dysfunction, resulting in urinary retention and subsequent pyelonephritis, particularly in immunocompromised patients. Careful monitoring of urinary symptoms and early intervention may help prevent serious infectious complications in this high-risk population.

## Introduction

For kidney transplant recipients, long-term immunosuppressive therapy is a risk factor for developing herpes zoster, and infections can potentially affect the function of the transplanted kidney [1]. Reports describing herpes zoster-associated urinary retention complicated by pyelonephritis in kidney transplant recipients remain limited.

Urinary retention associated with herpes zoster is uncommon but clinically important because delayed diagnosis and management may result in secondary urinary tract infections and upper urinary tract involvement. The use of immunosuppressive drugs after transplantation is the trigger. In addition, although urinary retention caused by herpes zoster involving the buttocks is relatively uncommon, it requires careful management because it may increase the risk of urinary tract infection [2].

We report a case of a kidney transplant recipient who developed acute urinary retention secondary to sacral herpes zoster involving the left buttock, subsequently leading to pyelonephritis of the transplanted kidney. This case highlights the importance of recognizing voiding dysfunction as a potential complication of herpes zoster in immunocompromised patients and the need for prompt urinary management to prevent severe infectious complications.

## Case presentation

The patient was a 59-year-old woman. She presented to our department with erythema and blisters on the left buttock, accompanied by difficulty urinating. Her medical history included chronic renal failure due to lupus nephritis, for which a living donor kidney transplant was performed 30 years ago. She did not have diabetes.

Following the transplant, she received immunosuppressive therapy with methylprednisolone 4 mg, mizoribine 10 mg, and cyclosporine 120 mg. Pain in the buttocks had started three days before the first visit. Erythema, blisters, and some pustules were found in the S2~S4 area of the left buttock at the time of the examination (Figure 1). DermaQuick® VZV (Maruho Co., Ltd., Osaka, Japan) was positive. The patient was diagnosed with sacral herpes zoster involving the left buttock, prescribed 400 mg/day of amenamevir orally, and went home. At that time, there were no urinary or bowel problems. However, two days later, the patient developed difficult urination and was admitted to the hospital after a follow-up visit the next day. The possibility of drug-induced urinary retention was ruled out.

**Figure 1:**
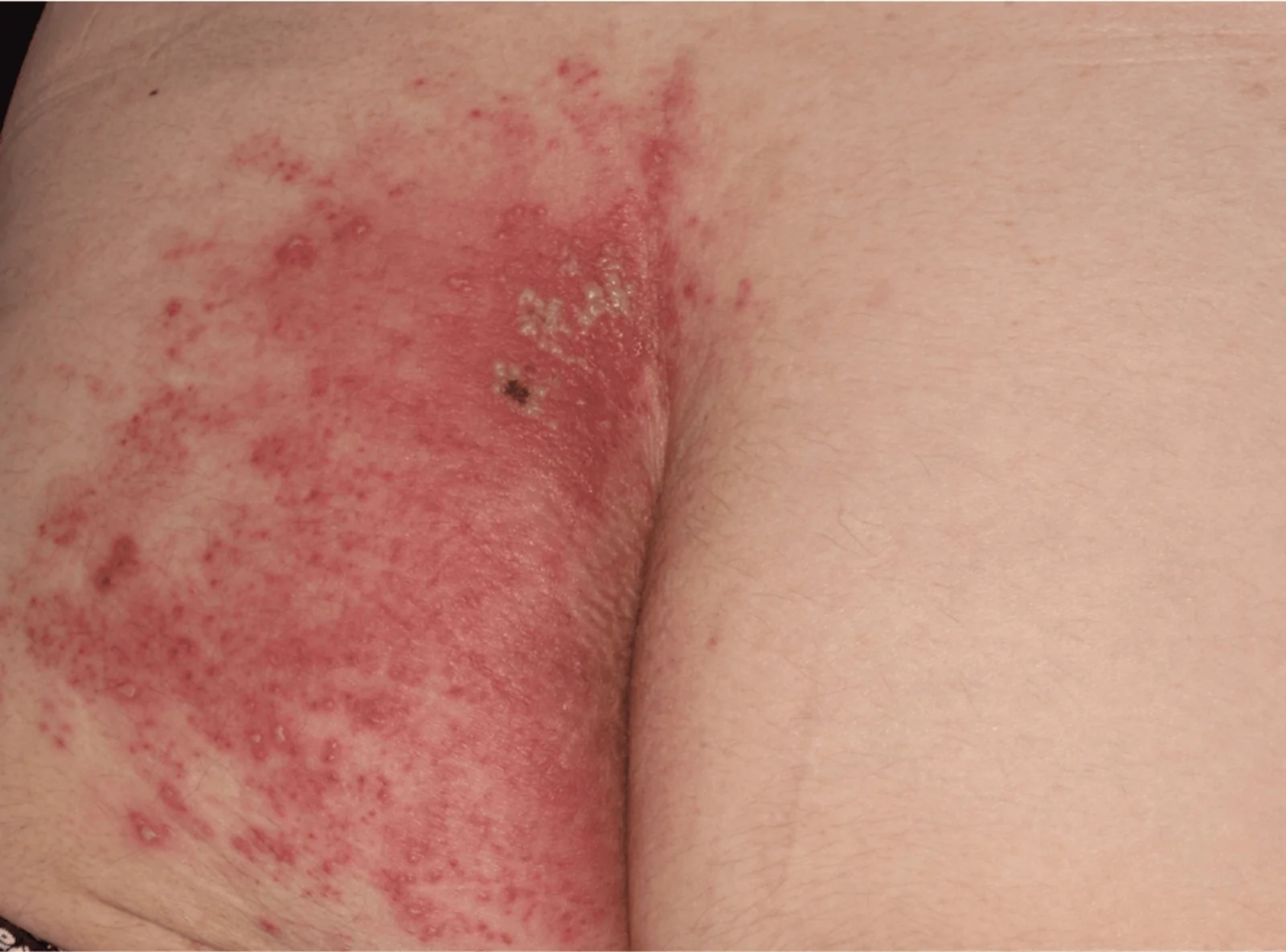
Sacral herpes zoster involving the left buttock.

Acyclovir was administered by intravenous infusion at a dose of 330 mg (5 mg/kg) three times daily for seven days, adjusted for her body weight and renal function. Regarding the difficulty in voiding observed the day before admission, intermittent catheterization was selected over an indwelling catheter because the patient had undergone a kidney transplant and intermittent catheterization carries a lower risk of infection. She complained of bladder fullness and pain, and 650 mL of urine was collected during the initial intermittent insertion of a urinary catheter. After the collection, the complaints of bladder fullness and pain subsided. Prednisone 60 mg was started on the first day of hospitalization, and after two intermittent catheterizations, the patient gradually became able to urinate on her own during the night, and by the fourth day, she was able to urinate normally, so the amount of prednisone was gradually reduced. Prednisolone was tapered as follows: 60 mg/day for three days, 30 mg/day for three days, 10 mg/day for four days, 5 mg/day for four days, and 3 mg/day for two days, and then discontinued. The rash gradually crusted over. During hospitalization, no bowel dysfunction was observed. Furthermore, no typical complications of herpes zoster, such as central nervous system symptoms, were observed. The pain from the shingles on the left buttock was almost completely gone by the second day of hospitalization. 

However, on the seventh day of her hospitalization, she had a fever of 38.5 degrees Celsius and tenderness at the site of the transplanted kidney in the right iliac fossa. Urine tests showed positive results for both white blood cell count and bacterial count, while blood tests revealed elevated white blood cell count, CRP, and creatinine levels (Table 1). *Escherichia coli* was detected in both blood cultures (two sets) and urine cultures. Native CT images revealed enlargement of the transplanted kidney and increased density of the perirenal adipose tissue (Figure 2). No findings suggestive of obstruction, hydronephrosis, or abscess were observed. Subsequently, the patient became afebrile, and blood tests showed improvement in creatinine levels; therefore, a follow-up CT scan was not performed. She was diagnosed with pyelonephritis and transferred to the Department of Nephrology for treatment (Figure 3). She was started on treatment with piperacillin/tazobactam 4.5 g administered three times daily for four days. Subsequently, after confirming drug susceptibility via culture testing, ampicillin was administered intravenously at a dose of 1 g three times daily for five days. On the 15^th^ day of hospitalization, improvements were observed in both urine and blood tests, and the creatinine level also decreased (Table 1).

**Table 1:** Blood and urine test reports of the patient. During hospitalization, blood tests revealed elevated levels of inflammation and creatinine. Additionally, urinalysis showed positive results for leukocytes and bacteria.

Parameter	Reference value	Before hospitalization	Hospital day 8	At discharge	Two weeks after discharge
Hematology					
White blood cell count (/μL)	3300～8600	4580	11680	5730	4190
C-reactive protein (mg/dL)	0.00～0.14	0.15	14.34	0.58	0.11
Potassium (mmol/L)	3.6～4.8	3.5	3.4	4.2	4.4
Urea nitrogen (mg/dL)	8.0～20.0	17	23	21	19
Creatinine (mg/dL)	0.46～0.79	0.89	1.25	0.94	0.99
Estimated glomerular filtration rate (mL/min/1.73m^2^)	-	50.5	34.8	47.6	45
Urinalysis					
Cloudy urine	Clear	Clear	Slightly cloudy	Clear	Clear
Leukocyte esterase	-	-	3＋	-	-
Bacteria	-	-	＋	-	-

**Figure 2:**
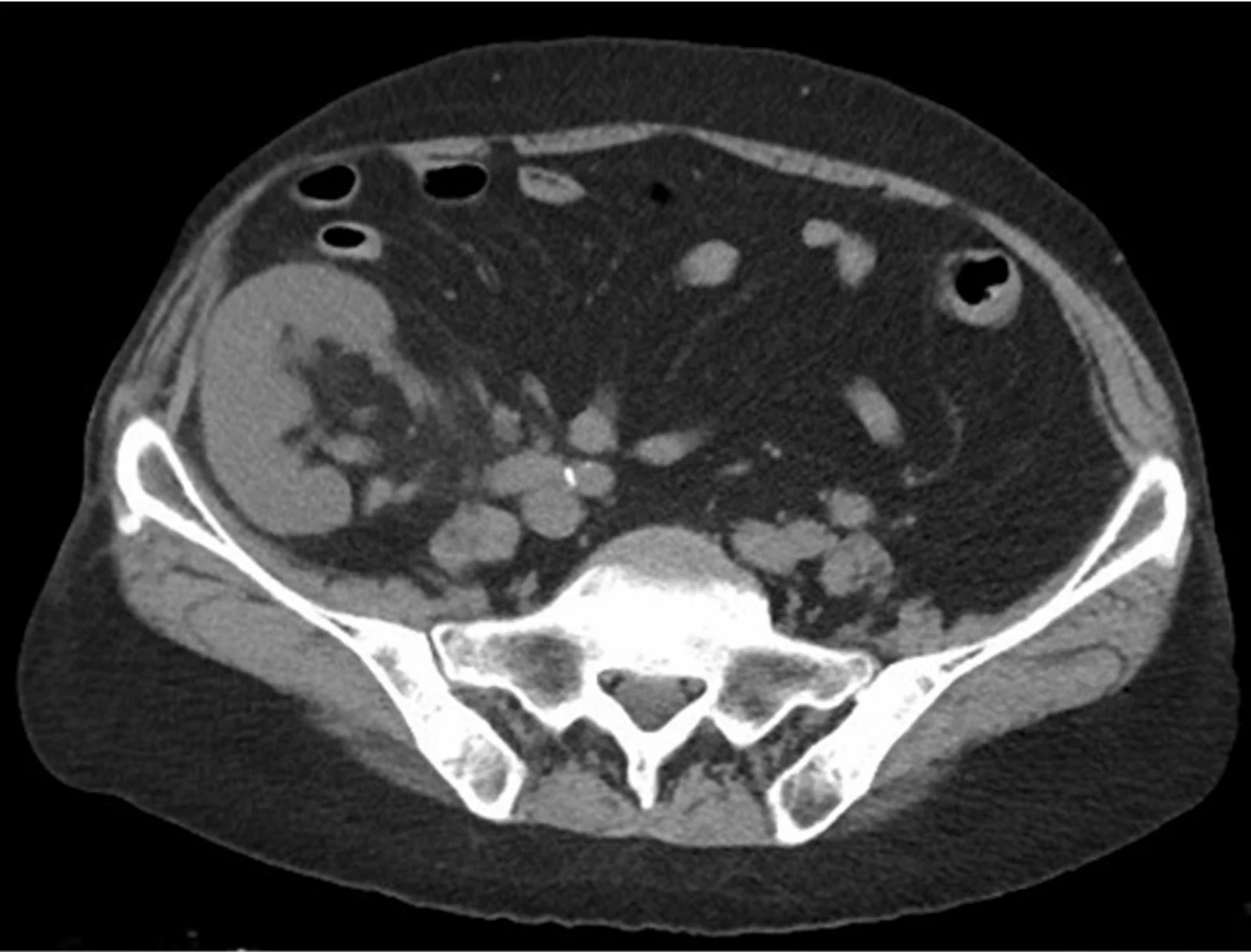
Native CT images revealed enlargement of the transplanted kidney and increased density of the perirenal adipose tissue. No findings suggestive of obstruction, hydronephrosis, or abscess were observed.

**Figure 3:**
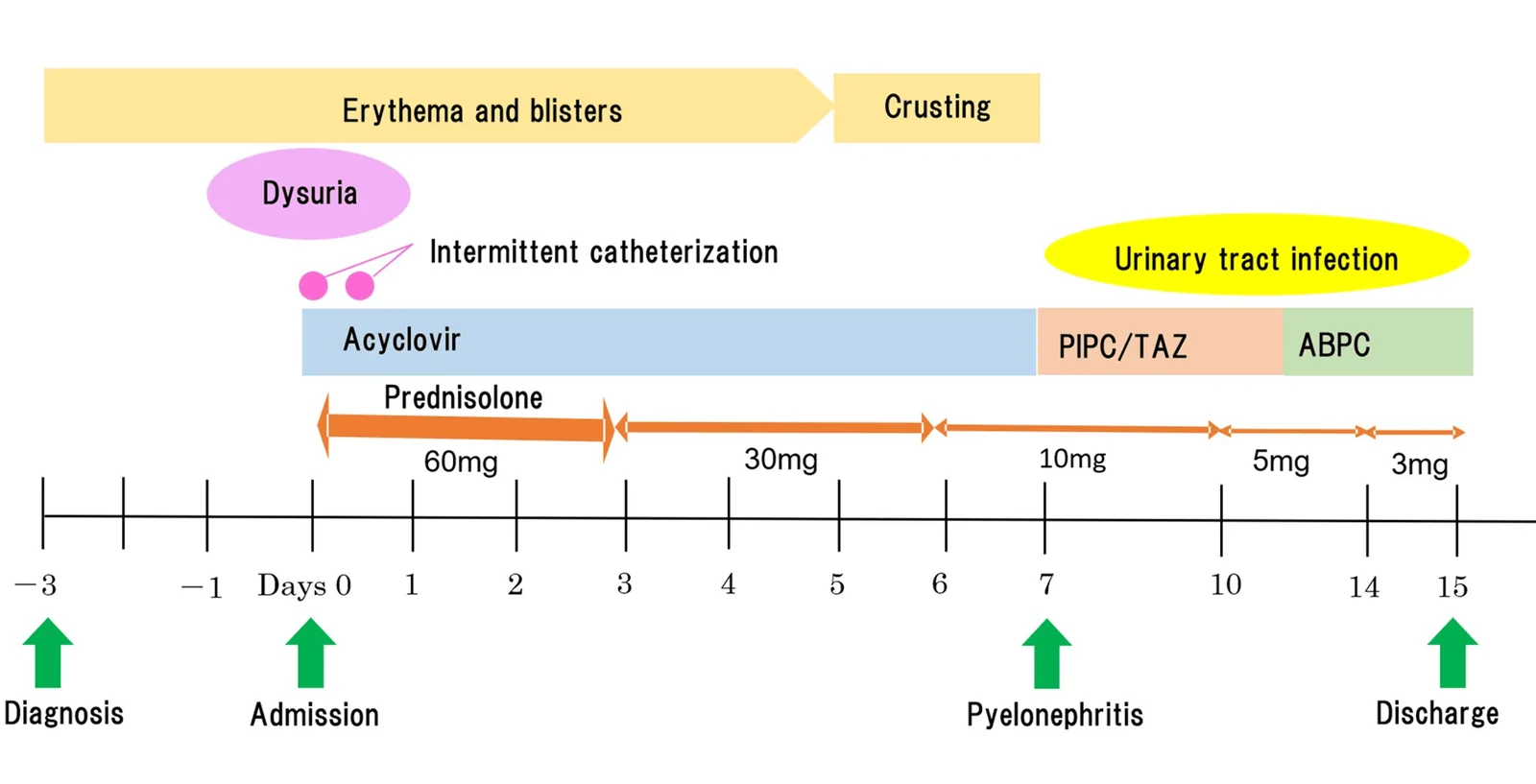
Clinical course in this case with clinical findings and medications. The patient was hospitalized and started receiving acyclovir infusion and prednisolone, and the urinary dysfunction improved. The prednisolone dosage was also gradually reduced. On the seventh day of hospitalization, the patient developed a fever and urinary tract problems, but these symptoms improved with antibiotic treatment. PIPC/TAZ: piperacillin/tazobactam; ABPC: ampicillin

## Discussion

Herpes zoster infection in the sacral pulp area accounts for about 4% of all herpes zoster infections [2]. Dysuria can occur not only with sacral herpes zoster but also with thoracolumbar herpes zoster [3]. The innervation of urethral sensation is mainly thought to be by the pudendal nerve (S2-S4), which explains the dysuria [3].

A total of 450 patients after renal transplantation were eligible, with a median follow-up of 38 months; 6.4% developed herpes zoster infection [4]. According to the latest report in Japan, 15.7% of kidney transplant patients had complications of herpes zoster infection [5]. The median age at onset was 51 years, and the median time from transplantation to onset was 7.8 years [5].

Kidney transplant patients are at increased risk of urinary tract infections due to immunosuppression, urinary tract anatomical changes, and complex comorbidities [6]. Specifically, the immune function against new infections decreases due to the decrease in systemic immunity caused by the administration of immunosuppressants. In kidney transplant surgery, narrowing of the urinary tract occurs due to the combination of the ureter and the donor's bladder. In addition, vesicoureteral reflux is more likely to occur. Transplant surgery cuts the surrounding nerves, which interferes with normal bladder function [6].

It has been reported that transplanted kidneys after pyelonephritis develop renal dysfunction [7]. In this case, there was a temporary increase in blood creatinine immediately after pyelonephritis. It is necessary to prevent pyelitis early.

Transient urination disorders such as urinary retention can cause urinary tract infections, which require caution [6]. While there are no established guidelines regarding the indications for or tapering schedules of corticosteroids for herpes zoster complicated by voiding dysfunction, reports indicate rapid improvement following early administration [8].

Systemic corticosteroids are the standard recommended treatment for facial nerve paralysis associated with Ramsay Hunt syndrome, and sporadic case reports describe their use in cases of herpes zoster complicated by voiding dysfunction. Clinicians must remain vigilant for potential adverse effects associated with corticosteroid use. However, regardless of whether corticosteroids are administered, most cases resolve within a few weeks through management with antiviral drugs and urethral catheterization, resulting in a favorable prognosis [8]. Further study of clinical cases is required to establish the efficacy of corticosteroid therapy and to determine optimal dosages and tapering regimens. This case highlights that herpes zoster involving the sacral or gluteal region can be associated with urinary dysfunction.

## Conclusions

In this case, the patient developed voiding dysfunction secondary to sacral herpes zoster following kidney transplantation. A subsequent urinary tract infection occurred, potentially linked to urinary retention. Caution is warranted, as temporary voiding difficulty caused by herpes zoster can lead to urinary tract infections.
